# Identifying important conservation areas for the clouded leopard *Neofelis nebulosa* in a mountainous landscape: Inference from spatial modeling techniques

**DOI:** 10.1002/ece3.3970

**Published:** 2018-04-02

**Authors:** Ugyen Penjor, David W. Macdonald, Sonam Wangchuk, Tandin Tandin, Cedric Kai Wei Tan

**Affiliations:** ^1^ Wildlife Conservation Research Unit Department of Zoology University of Oxford, The Recanati‐Kaplan Centre Tubney Oxfordshire UK; ^2^ Nature Conservation Division Department of Forests and Park Services Ministry of Agriculture and Forests Thimphu Bhutan

**Keywords:** conservation planning, density, occupancy models, site use, spatial autocorrelation

## Abstract

The survival of large carnivores is increasingly precarious due to extensive human development that causes the habitat loss and fragmentation. Habitat selection is influenced by anthropogenic as well as environmental factors, and understanding these relationships is important for conservation management. We assessed the environmental and anthropogenic variables that influence site use of clouded leopard *Neofelis nebulosa* in Bhutan, estimated their population density, and used the results to predict the species’ site use across Bhutan. We used a large camera‐trap dataset from the national tiger survey to estimate for clouded leopards, for the first time in Bhutan, (1) population density using spatially explicit capture–recapture models and (2) site‐use probability using occupancy models accounting for spatial autocorrelation. Population density was estimated at ${\hat{D}}_{\mathrm{Bayesian}}=0.40$ (0.10 *SD*) and ${\hat{D}}_{\mathrm{maximum}-\mathrm{likelihood}}=0.30$ (0.12 *SE*) per 100 km^2^. Clouded leopard site use was positively associated with forest cover and distance to river while negatively associated with elevation. Mean site‐use probability (from the Bayesian spatial model) was ${\hat{\psi }}_{\mathrm{spatial}}=0.448$ (0.076 *SD*). When spatial autocorrelation was ignored, the probability of site use was overestimated, ${\hat{\psi }}_{\mathrm{nonspatial}}=0.826$ (0.066 *SD*). Predictive mapping allowed us to identify important conservation areas and priority habitats to sustain the future of these elusive, ambassador felids and associated guilds. Multiple sites in the south, many of them outside of protected areas, were identified as habitats suitable for this species, adding evidence to conservation planning for clouded leopards in continental South Asia.

## 1 INTRODUCTION

Understanding species’ distributions and their responses to environmental factors and anthropogenic influences are important aspects of conservation planning (Block & Brennan, 1993; MacKenzie et al., 2006). Most management plans for endangered species are based on species–environment relationships and species range maps, and understanding the factors determining site suitability is an important element of the landscape approach to carnivore conservation (Chapron et al., 2014). For example, proportion of area occupied (PAO) and extent of occurrence (EOO) are used extensively to calculate species distributions in the IUCN Red List (IUCN, 2001), thus enabling the identification of important areas for conservation management and protection. In addition, estimating reliable population density is critical in animal ecology as inferences on the population dynamics of species that naturally occur in low densities (such as most felids) are central to their effective conservation and management (Karanth, Nichols, Kumar, & Hines, 2006; Kéry, Gardner, Stoeckle, Weber, & Royle, 2010; Obbard, Howe, & Kyle, 2010). Density and habitat‐use information are also important in assessing risk under climate, environmental, and land‐use scenarios (Dormann et al., 2007).

**Figure 1:**
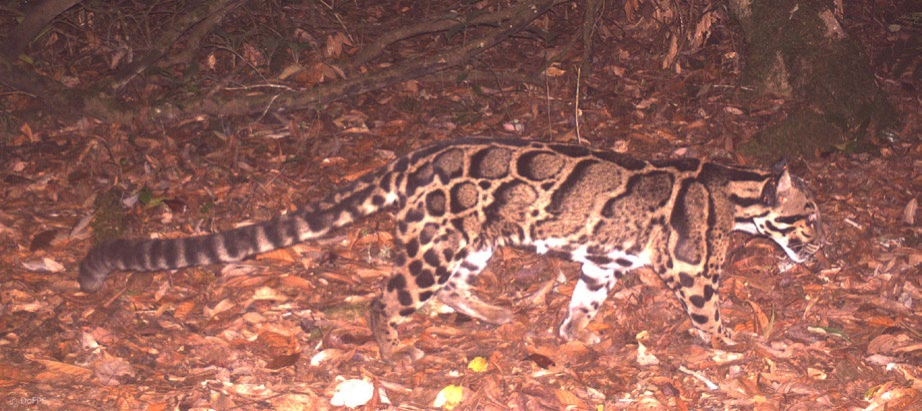
Clouded leopard *Neofelis nebulosa* captured in one of the camera traps in the study area

The clouded leopard *Neofelis nebulosa* is the smallest of the large felids (Austin, Tewes, Grassman, & Silvy, 2007; Figure 1). It is classified as Vulnerable by the International Union for Conservation of Nature (IUCN) Red List of Threatened Species and is included in Appendix I of the Convention on International Trade in Endangered Species of Wild Flora and Fauna (CITES; Grassman et al., 2016). The species is not well known, and the rapidity of environmental change in Bhutan adds urgency to acquiring ecological data to inform its conservation (Penjor, 2016).

Illegal persecution and trade in skins and parts threaten clouded leopard survival (Nowell & Jackson, 1996; Nijman & Shepherd, 2015; Min et al., 2018). Rapid development, increasing human population, deforestation and habitat fragmentation, and ineffective protected area management add to the pressing threats to the clouded leopard across its range (Choudhury, 1993; D'Cruze & Macdonald, 2015; DeFries, Hansen, Newton, & Hansen, 2005; Rabinowitz, 1988). Against this background, a reliable estimate of the population is an important datum for the understanding of forest carnivore guilds in Southeast Asia and also important to underpin conservation planning (Murphy & Noon, 1992).

There are two species of clouded leopards: *N. nebulosa* (mainland) distributed from Nepal to Peninsular Malaysia and *N. diardi* (Sunda) restricted to the islands of Sumatra and Borneo (Wilting et al.*,* 2007). Until the mid‐2000s, information on the clouded leopard in the wild was scant (Grassman, Tewes, Silvy, & Kreetiyutanont, 2005) and mostly came from reports, anecdotes, and captive animals (Rabinowitz, 1988; Yamada & Durrant, 1989 for *N. nebulosa*; Rabinowitz, Andau, & Chai, 1987 for *N. diardi*). In the late 1980s and early 1990s, information was gathered through local sightings and survey reports across the distribution range of the two species of clouded leopard (e.g., Borneo, Rabinowitz et al., 1987; Nepal, Dinerstein & Mehta, 1989; Thailand, Davies, 1990; India, Choudhury, 1993; Taiwan, Rabinowitz, 1988; for *N. nebulosa*; Sumatra, Santiapillai & Ashby, 1988; for *N. diardi*). The only quantitative information available prior to 2006 was provided by a pugmark survey by Davies and Payne (1982; for *N. nebulosa*). Since 2006, there has been an upsurge in studies estimating habitat use (Ngoprasert et al., 2012; in Thailand; Mohamad et al., 2015; and Tan et al., 2017; in Peninsular Malaysia for *N. nebulosa*; Haidir, Dinata, Linkie, & Macdonald, 2013; in Sumatra; Sunarto, Kelly, Parakkasi, & Hutajulu, 2015; in Sumatra for *N. diardi*) and density (Borah et al.*,* 2014, in India for *N. nebulosa*; Wilting, Fischer, Bakar, & Linsenmair, 2006; in Sabah, Malaysia; Wilting et al., 2012; and Brodie & Giordano, 2012; in Sabah, Borneo; Cheyne, Stark, Limin, & Macdonald, 2013; in Indonesia; Sollmann, Linkie, Haidir, & Macdonald, 2014; in Sumatra for *N. diardi*). These density estimation studies used widely accepted hierarchical modeling and spatially explicit methods (Table S6). Three studies, part of our rangewide study, have recently provided substantial information (Tan et al., 2017; Peninsular Malaysia; Singh & Macdonald, 2017; India; Naing, Ross, Burnham, Htun, & Macdonald, 2017; Myanmar). In Bhutan, there is no baseline information on clouded leopard numbers or threats (Penjor, 2016) despite it being protected under stringent legislation (RGoB, 1995). In 2013–2014, three cases involving the illegal poaching of clouded leopard were recorded in Bhutan (DoFPS, 2015) raising concerns that current legislation and protection effort are inadequate for conservation management. More broadly, development is rapid in Bhutan and forest loss is accelerating faster than expected (DoFPS, 2011).

We quantified the site use of clouded leopards using spatial occupancy models that account for spatial autocorrelation (SAC hereafter; Johnson, Conn, Hooten, Ray, & Pond, 2013) and for imperfect detection (MacKenzie et al., 2002, 2006). The spatial occupancy model partitions out the spatial component from the environmental effects. This approach improves inference when observations are spatially autocorrelated and helps to reduce bias and overestimated precision when observations are not conditionally independent given the covariates (Johnson et al., 2013). Although MacKenzie et al., (2009) state that using appropriate covariates would account for SAC, we decided to include a spatial component in our model because of nonindependence between camera stations (the same individual clouded leopards were “captured” at multiple stations) and correlated variables (Broms, Johnson, Altwegg, & Conquest, 2014; Dormann et al., 2007; Johnson et al., 2013). Density is estimated using both maximum‐likelihood (Efford et al., 2009) and Bayesian (Royle, Chandler, Sollmann, & Gardner, 2014) models, enabling us to compare the estimates from the two (Noss et al., 2012). These spatial capture–recapture (SCR) models incorporate spatial information on detection and allow for heterogeneity based on sex, age, and response behavior of animals (Royle et al., 2014). These improve reliability of the density estimates when compared to nonspatial models (Efford et al., 2009).

This study used data from the national tiger survey, the first comprehensive nationwide camera‐trapping survey conducted to assess the tiger population in Bhutan. We aimed to (1) provide the first population density estimate of the clouded leopard in Bhutan, (2) identify the environmental and anthropogenic variables that influence site use, and (3) predict the site‐use intensity across Bhutan and identify important areas for protection. We hypothesized that clouded leopards would prefer forested habitat away from anthropogenic features such as settlements and roads (Austin et al., 2007; Sunarto et al., 2012).

## 2 MATERIALS AND METHODS

### 2.1 Study area

**Figure 2:**
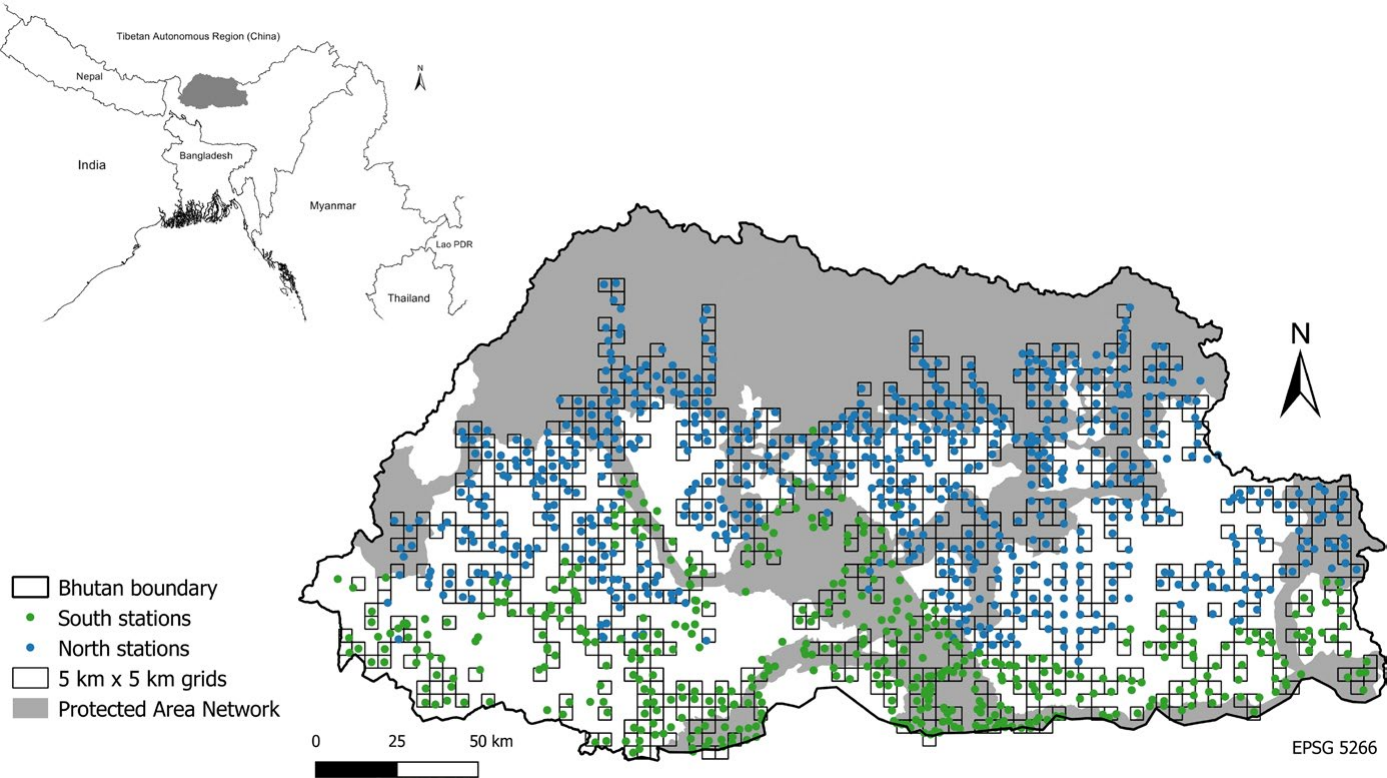
Location of Bhutan in continental Southeast Asia and camera locations

Bhutan (27°24′14″N, 90°24′2″E) is a small landlocked country bordered by the Tibetan Autonomous Region (China) in the north and India in the east, west, and south (Figure 2). The area of the country is 38,394 km^2^ with a total population of 779,666 (NSB, 2017). The climate is characterized by four distinct seasons: winter (December–February), spring (March–May), summer (June–August), and autumn (September–November). Rainfall is persistent and heavy during the monsoon (July–September), largely fed by moisture‐laden winds from the Bay of Bengal. Precipitation varies between 300 mm (north) and 5,000 mm (south) per year, and the temperature ranges from subzero in the north to above 35°C in the humid subtropical south. As a result, the vegetation type varies from lush subtropical broadleaved forest to dry alpine meadow as the altitude rises from <100 to >5,000 m a.s.l.

### 2.2 Field survey

The survey was organized in two major blocks: south and north. It was conducted between March 2014 and March 2015. The survey was not conducted at settlements, agriculture land, and areas above 4,500 m because we judged these areas as nonhabitat and there was no previous record of clouded leopard reported at these places. After eliminations of these areas, a grid of 1,522 grid cells and 25‐km^2^ grid space was laid out across the country (Figure 2; for details of survey area refer to Table 3). In total, 1,129 (*n*_north_ = 681; *n*_south_ = 448) camera stations were deployed along animal tracks or locations where field signs suggested maximum detection probability. Five different camera models (Bushnell^™^, CuddeBack^™^, HCO‐ScountGuard^™^, Reconyx^™^, and U‐Way^™^) were used for the survey. In each station, two cameras were installed, 45 cm above the ground and at least 5 m apart, facing each other. A minimum distance of 2 km between each station was maintained whenever possible in the context of impassable rivers, steep slopes, and settlements. The camera traps were deployed for four months in the south (March–June 2014) and 5 months in the north (October 2014–March 2015). Monthly checks were carried out to retrieve data, change batteries, and clear obstacles in front of the camera lenses.

We used images from stations that captured both flanks of clouded leopard to estimate their density. At some stations, the clouded leopard was captured at only one camera trap. Such single images cannot be identified as individuals and so were excluded from the density estimation but not the occupancy analysis (Foster & Harmsen, 2012). We used data from the south block only to estimate clouded leopard population density because the sample size was adequate (*n*_south_ = 448) and the cameras were deployed on a single season (March–June 2014). The number of captures in the north block was few (9 of 681 stations), and while this is a revealing result in itself, the numbers were too low for density estimation (Figure S2). We used the entire sample to analyze site‐use probability (*n*_total_ = 849).

### 2.3 Data analysis

#### 2.3.1 Site use

The photographic records of clouded leopard were converted into binary detection histories representing 1 for detection and 0 for nondetection (MacKenzie et al., 2002). To minimize the risk of violating the closure assumption when estimating site use, the capture period used for the analysis was the first 120 days of each camera station's history (Rota, Fletcher, Dorazio, & Betts, 2009). Further, only one survey had deployment >120 days (Table S7). The 120‐day subset was collapsed into sampling periods to increase temporal independence among occasions and overall detection probability (Dillon & Kelly, 2007; Otis, Burnham, White, & Anderson, 1978; Tan et al., 2017). The optimum number of days per occasion was selected based on chi‐square goodness‐of‐fit test for the multivariate global model (MacKenzie & Bailey, 2004). A 15‐day period proved optimal and was used for further occupancy analyses (Table S3).

Occupancy ψ is defined as the probability that a species will occupy a random site at a given time period (MacKenzie et al., 2006). We refer to ψ as the probability of site use (MacKenzie et al., 2006, p. 105), which is the probability of clouded leopard using a sampling unit (camera station; Mohamad et al., 2015). Occupancy models can accommodate covariates, and hence, detection and occupancy probabilities can be modeled as a function of a survey and site‐specific covariates (MacKenzie et al., 2006). Site covariates were selected based on literature on clouded leopards (Mohamad et al., 2015; Tan et al., 2017). Site covariates for the whole of Bhutan were processed using QGIS 2.14 (QGIS Development Team, 2016) and ArcGIS 10.2 (ESRI, 2011). Each site covariate value was the mean of raster cells bound within concentric circles of 500 m radius around each camera‐trap station. It was calculated using the “zonal statistics” tool in QGIS. This radius distance was chosen to represent the average site characteristics around each camera station. Elevation, slope, and aspect values were extracted from a 30‐m resolution digital elevation model raster data (DEM; USGS, 2016). Vegetation data came from two different sources: (1) a 250‐m resolution vegetation continuous field (VCF) which shows the percentage of pixels covered by vegetation above 5 m height (DiMiceli et al., 2011) and (2) a 30‐m resolution global forest change (GFC) cover for the year 2014 (the camera traps were deployed in that year) (Hansen et al., 2013). With the GFC layer, users can define the percentage of tree cover to be considered as forest. GFC thresholds of 30%, 50%, 75%, and 90% tree cover were tested independently for their effects on occupancy via univariate modeling (Table S2). The different threshold levels were chosen to avoid subjective selection as we did not have supplementary data or field verification (Tan et al., 2017; Tropek et al., 2013). Distance to major rivers, settlements, roads, protected areas, and commercially logged forest was generated using the Euclidean distance tool in ArcGIS. These vector layers were rasterized to generate the distances in meters.

We ran single‐season, single‐species occupancy models with the R package “unmarked” (Fiske & Chandler, 2011) to estimate the maximum‐likelihood probability of site use, accounting for imperfect detection (MacKenzie et al., 2006). Comparison between all possible models of covariates was made using the R package “AICcmodavg” (Mazerolle, 2015). We used AIC_c_ corrected for small sample size (AIC_c_) for model selection (Burnham & Anderson, 2004). All multivariate models within ΔAIC_c_ < 2 of the best‐performing multivariate models were considered to be strongly supported by the data (Burnham & Anderson, 2004). The parameter estimate of each covariate was averaged using the r package “MuMIn” (Barton, 2016).

Detection probability *p* was modeled as a function of survey covariates viz., survey area and effort (number of active camera‐trap days during each sampling occasion). Site‐use probability ψ was modeled as a function of site covariates (aspect, elevation, distance to logged forest, GFC and VCF forest cover, distance to river, distance to road, distance to protected area, distance to settlement, and slope). A two‐stage modeling approach was adopted to reduce the number of combinations of every possible covariate (Long, Donovan, MacKay, Zielinski, & Buzas, 2011). First, we modeled the detection *p*(.), while keeping ψ(.) constant by running possible combinations for detection covariates (effort and survey area) additively, that is, *p*(variable) ψ(.). Probabilities of detection might differ among survey areas, which could be due to various factors such as disturbance level in the survey area or topography (Tan et al., 2017). We then retained significant detection covariates and ran multivariate models for occupancy with site covariates.

All continuous site covariates were z‐standardized $x-\overset{¯}{x}/\sigma$ to a mean of 0 and unit standard deviation prior to analysis in order to facilitate model convergence and comparisons among covariates (Stanton, Thompson, & Kesler, 2015). A preliminary set of 13 covariates was tested for collinearity using Pearson's correlation in R 3.3.1 (R Core Team, 2016). Any pairwise combination with coefficient |*r*| ≥ 0.6 was considered correlated (Table S1). Multivariate collinearity among predictor variables is often found to hinder the analysis of ecological processes, thus producing biased estimates (Cade, 2015). Therefore, from the correlated pairs, the covariate that performed better (low AIC_c_) in the univariate single‐season occupancy models was retained for the multivariate model. The goodness‐of‐fit test for the most parameterized multivariate model was performed with 1,000 bootstraps (MacKenzie & Bailey, 2004).

To account for SAC in the occupancy models, we used R package “stocc” (Johnson, 2015) to perform the spatial occupancy modeling of site use (Johnson et al., 2013). The best model from the maximum‐likelihood analysis was fitted using a Bayesian framework following Broms et al. (2014; Kéry & Schaub, 2012). This method employs restricted spatial regression (RSR) using the *probit* link formulation (Johnson et al., 2013). RSR improves computational efficiency, minimizes confounding between parameters, and corrects for spatial effects in the covariates (see Johnson et al., 2013; and Broms et al., 2014, for details). The final model describing the site‐use probability was

$$\begin{array}{cc} \text{probit}(\psi_{i})= & {\hat{\beta }}_{0}+{\hat{\beta }}_{\mathrm{ele}}{\text{elevation}}_{i}+{\hat{\beta }}_{\mathrm{for}}\text{forest}\;{\text{cover}}_{i}\\ & +{\hat{\beta }}_{\mathrm{riv}}\text{distance to}\;{\text{river}}_{i}+\eta_{i(\tau )} \end{array}$$

where ${\hat{\beta }}_{0}$ refers to intercept, ${\hat{\beta }}_{i}$ are the coefficient estimates of the covariates, *i* is the site surveyed, η_*i*(τ)_ represents the spatial process for the *i*th site, and τ ~ Gamma (0.5, 0.0005) is a parameter that controls the spatial process. Following Johnson et al. (2013), the prior for the τ heavily weights large value (implying less spatial autocorrelation). As a result, any spatial effect seen is strong evidence for spatial autocorrelation.

The Moran cut used in the spatial model was 10% of the total number of camera stations (0.1 × 849 = 84.9; Hughes & Haran, 2010). The Moran statistic is a measure of SAC, analogously interpreted as the correlation coefficient for the correlation in site‐use probabilities across sites (see Johnson et al., 2013; for details). The threshold of 8.7 km was used. This indicates that all sites within this distance are considered neighbors and this distance was chosen based on the movement parameter derived from spatial capture–recapture analysis (see 3.2 estimates in the Section 4; Table 4). Each Bayesian model was run with 1 chain for 50,000 iterations. We discarded the first 10,000 as burn‐in and employed a thinning rate of 5.

The mean untransformed beta coefficients and 95% credible intervals (CRI) were used to examine the degree and direction of the covariate effect on the site‐use probability. We considered covariates to have a strong influence on the clouded leopard's site use if their 95% CRI did not include 0.

We used coefficient estimates from the Bayesian model corrected for SAC to predict clouded leopard site use in forested pixels across Bhutan. Univariate modeling revealed that GFC (forest cover) was an important variable influencing clouded leopard site use. For each forested pixel (for the year 2014), we extracted values of important covariates using a 90‐m resolution. We then predicted site use for these pixels with parameter estimates from the best multivariate model. These pixels were then imported into QGIS to map out clouded leopard site suitability across Bhutan.

#### 2.3.2 Density

Density was estimated using maximum‐likelihood and Bayesian approaches both of which are spatially explicit capture–recapture (SCR) methods (Efford et al., 2009; Royle et al., 2014). We chose to apply both methods to the same dataset for comparison (Noss et al., 2012). Spatial methods address edge effects using temporal and spatial information in addition to detection data. Further, they eliminate ad hoc estimation of the effective survey area (Efford, Borchers, & Byrom, 2009; Noss et al., 2012). The R package “secr” (Efford, 2016) was used to estimate maximum‐likelihood density (Efford et al., 2009), and the package “SPACECAP” (Gopalaswamy et al., 2014) was used to estimate density using a Bayesian modeling framework. It is assumed that animals have circular home ranges with fixed centers (Royle et al., 2014). The half‐normal detection function was used to model home range, and this is comprised of two parameters: *g*_0_ *,* the encounter rate at the home range centre, and the scale parameter σ*,* which describes the decline of the encounter rate from home range centers (Tobler & Powell, 2013).

A spatial mask comprising of a 25‐km buffer was created around the outermost camera‐trap stations (Soisalo & Cavalcanti, 2006). This buffer distance corresponds to approximately 3× the scale parameter (σ) (8.7 km) and is large enough to contain potential home range centers of all clouded leopards caught on our survey grid. All unsuitable habitats, such as settlements, roads, and large rivers, were assigned 0 as opposed to suitable habitats (assigned 1). Equidistant points in the space mask represented potential activity centers. The spacing between points was entered as 500 m. This is the shortest distance allowable and enables fine‐scale modeling of where actual home range centers might be based on the camera‐trap data (Efford & Efford, 2016; Gopalaswamy et al., 2014). For the SPACECAP analysis, the input data were prepared as prescribed in the SPACECAP manual (Gopalaswamy et al., 2014). We executed 60,000 Markov chain Monte Carlo (MCMC) iterations, a burn‐in of 10,000, and a thinning rate of 1. The data augmentation value was set to 190 (10 times the number of the total number of identified individuals). Geweke diagnostic scores were used to check convergence of chains (Gopalaswamy et al., 2012). We report posterior means with standard deviations and 95% highest posterior density intervals (HPDI). Due to ambiguity in photographs, sex‐based identification was not possible; thus, we did not report the sex‐specific density estimates in both approaches.

A surface density map was generated to depict posterior estimates of pixel densities. The final discrete habitat map consisted of equally spaced grid cells of 0.25 km^2^, for visualizing the fine‐scale variation in density across southern Bhutan (Figure S3).

## 3 RESULTS

We retrieved camera traps from 849 stations (of 1,129 stations), the rest were lost to animal damage, theft, and malfunction. All stations in the south were recovered (448 of 448), while only 401 of 681 stations were recovered from the northern block.

### 3.1 Site use

Clouded leopards were detected in 114 of 849 stations totaling an effort of 62,739 trap days and giving the overall naïve occupancy estimate of 0.134. All the GFC forest cover variables were correlated with one another and with VCF. Within each correlated pair, the covariate performing better in the univariate occupancy models were GFC at 90% threshold (GFC90), elevation (ELE), slope (SLO), aspect (ASP), distance to logged forest (LOG), distance to major river (RIV), distance to protected area (PA), distance to road (ROA), and distance to settlement (SET). MacKenzie and Bailey (2004) goodness‐of‐fit test of the most parameterized multivariate model with uncorrelated covariates showed no evidence of overdispersion (*ĉ* = 0.74, χ^2^ = 872.70, *p* = .206).

The probability of detection was influenced by survey area and effort (number of active camera‐trap days per sampling occasion; Table 1). The effort was positively correlated with detection probability $\hat{\beta }\left(\hat{\mathrm{SE}}\right)=0.085$ (0.023) and varied across the surveyed areas (Table 3; Figure S4). The model with elevation, forest cover, and distance to river had the highest support from the likelihood‐based analysis (Table 2). As for the Bayesian analyses, a slightly lower posterior predictive loss criterion was reported for the spatial model as compared to the nonspatial model (PPLC_spatial_ = 316.806; PPLC_nonspatial_ = 317.085). This indicates that the inclusion of the spatial random effect could be necessary. Similarly, for the spatial model, the posterior distribution of the spatial variance parameter ($\sigma =1/\sqrt{\tau }$) was not very far from zero (95% CRI: 0.053–34,104.68), implying that additional spatial effect contributed to the variability of site use (Johnson et al., 2013) but not to a large extent. The spatial occupancy model resulted in lower site‐use probability and narrower CRI compared to likelihood‐based estimates (${\hat{\psi }}_{\mathrm{nonspatial}}=0.829$ (0.066 *SE*) versus ${\hat{\psi }}_{\mathrm{spatial}}=0.448$ (0.076 *SE*); Table 3, Figure 4). There was strong evidence to suggest that site use was negatively associated with elevation and positively associated with forest cover and distance to rivers as the 95% CRIs did not include zero (Table 3). The mean predicted site‐use probability in forested areas is 0.448 (0.076 *SE*). Distance to logged forests and protected areas were negatively associated with site‐use probability, while site‐use probability was positively associated with distance to road, slope, and aspect. However, these effects were at best weak, with high standard errors and 95% CIs overlapping zero (Table S4).

**Table 1:** Detection probability (*p*) models

Model	AIC_c_	ΔAIC_c_	AIC_c_Wt	−2logLik	*K*
*p* (Survey Area + Effort)	1,288.50	0.00	1.00	−635.14	9
*p* (Survey Area)	1,304.99	16.49	0.00	−644.41	8
*p* (Effort)	1,351.64	63.14	0.00	−672.81	3
*p*(.)	1,372.91	84.41	0.00	−684.45	2

Covariates are different surveyed areas (Survey Area) and total number of active camera‐trap days (Effort). AIC_c_, Akaike information criterion corrected for small sample size; ΔAIC_c_, relative difference between AIC_c_ of subsequent models compared to the top model; AIC_c_Wt, AIC_c_ weight and K, number of parameters. Occupancy was held constant ψ(.).

**Table 2:** Multivariate model selection results of clouded leopard site‐use probability

Model	AIC_c_	ΔAIC_c_	AIC_c_Wt	−2logLik	*K*
ψ(ELE + GFC90 + RIV)	1,271.55	0.00	0.23	−623.59	12
ψ(ELE + GFC90 + LOG + RIV + ROA)	1,272.35	0.80	0.15	−621.92	14
ψ(ELE + GFC90 + RIV + ROA)	1,272.47	0.92	0.14	−623.02	13
ψ(ELE + GFC90 + RIV + SLO)	1,272.51	0.96	0.14	−623.04	13
ψ(ELE + GFC90 + LOG + RIV)	1,272.70	1.15	0.13	−623.13	13
ψ(ELE + GFC90 + PA + RIV)	1,272.80	1.25	0.12	−623.18	13
ψ(ASP + ELE + GFC90 + RIV)	1,272.54	1.99	0.08	−623.55	13
ψ(NULL)	1,372.91	92.22	0.00	−684.45	2

Models strongly supported by the data (ΔAIC_c_ < 2) are shown. Site covariates tested: elevation (ELE), global forest change of 90% tree cover threshold (GFC90), slope (SLO), aspect (ASP), distance to rivers (RIV), to logged forest (LOG), to road (ROA), to settlement (SET), and to protected area (PA). All models include different survey areas (Survey Area) and the number of active camera days per sampling occasion (Effort) as detection covariates, *p*(Survey Area + Effort).

**Table 3:** Parameter estimates, standard errors, and 95% CRIs from the best‐fitting model for clouded leopard site use

	Covariates	Maximum‐likelihood (*logit* scale)			Bayesian (*probit* scale)		
		Nonspatial			Spatial		
		Mean	*SE*	95% CI^a^	Mean	*SD*	95% CRI^b^
*p*	Intercept	−6.716	1.28	**(**−**9.239,** −**4.202)**	−3.59	0.57	**(**−**4.784,** −**2.563)**
	Survey Area 2	0.953	1.17	(−1.338, 3.244)	0.493	0.51	(−0.431, 1.587)
	Survey Area 3	2.253	1.08	**(0.145, 4.361)**	1.108	0.47	**(0.27, 2.141)**
	Survey Area 4	2.975	1.06	**(0.890, 5.060)**	1.519	0.46	**(0.702, 2.535)**
	Survey Area 5	1.49	1.09	(−0.642, 3.623)	0.765	0.48	(−0.077, 1.804)
	Survey Area 6	2.424	1.09	**(0.292, 4.556)**	1.205	0.48	**(0.339, 2.259)**
	Survey Area 7	1.976	1.09	(−0.150, 4.102)	0.985	0.48	**(0.149, 2.020)**
	Effort	0.163	0.05	**(0.066**,** 0.261)**	0.085	0.02	**(0.042, 0.134)**
ψ	Intercept	−1.034	0.25	**(**−**1.520,** −**0.548)**	−0.47	0.14	**(**−**0.748,** −**0.187)**
	Elevation	−0.419	0.2	**(**−**0.803,** −**0.034)**	−0.244	0.11	**(**−**0.466,** −**0.038)**
	GFC90	2.647	1.11	**(0.472, 4.822)**	0.235	0.09	**(0.067, 0.427)**
	River	0.406	0.14	**(0.132, 0.681)**	0.265	0.09	**(0.098, 0.451)**

Site covariates tested: elevation (ELE), distance to river (RIV), global forest change 90% threshold (GFC90). Bold CRI indicates zero is not within the interval. Survey Area refers to different surveyed area where Survey Area 1: (Intercept) Samtse, Paro, Jigme Khesar Strict Nature Reserve; Survey Area 2: Jigme Dorji National Park, Thimphu, Wangdue; Survey Area 3: Gedu, Tsirang, Phipsoo Wildlife Sanctuary; Survey Area 4: Sarpang, Jigme Singye Wangchuck National Park, Royal Manas National Park; Survey Area 5: Wangchuck Centennial National Park, Bumthang, Zhemgang; Survey Area 6: Mongar, Phrumsengla National Park, Bumdelling Wildlife Sanctuary; and Survey Area 7: Trashigang, Samdrupjongkhar, Sakteng Wildlife Sanctuary.

a Confidence interval.

b Credible interval.

In estimating site‐use probabilities, we found that the mean site‐use probabilities of protected and nonprotected areas were similar (${\hat{\psi }}_{\mathrm{PA}}=0.431$(0.056 *SE*); ${\hat{\psi }}_{\mathrm{outside}\mathrm{PA}}=0.451$ (0.060 *SE*)). Sites inside and outside of protected areas in the southern region were predicted to have higher use probability than sites inside and outside protected areas in the north (Figure 4; Figure S1).

### 3.2 Density

We used 321 images and 20 videos from south stations only (*n*_stations_ = 448) for the year 2014 for SCR analysis. The total effort was 23,249 trap days. The mean spacing between the successful stations was 2,963 m (±1,238 m *SD*); however, the distance between all stations was not uniform due to terrain (range: 80–16,600 m). Nineteen individuals were identified. The number of captures and recaptures ranged from one to three for identified individuals (mean ±*SD* = 1.37 ± 0.58; Figure S2).

Results from the Bayesian analysis are presented in Table 4. Estimated clouded leopard density in south Bhutan was 0.40 (±0.10 *SD*) per 100 km^2^. We present the key parameters of baseline encounter rate (λ_0_) of clouded leopard individuals at the stations, the scale parameter (σ), and density in the space state (*D*). Geweke diagnostic scores showed model convergence <±1.6 (λ_0_ = −1.12, σ = −0.53, ψ = 1.13, *N*_super_ = 1.13). Further, Bayesian *p*‐value (=.47) indicates that our model adequately described the data. Abundance throughout the effective sampled area (ca. 20,500 km^2^) is estimated at 80 (±32 *SD*) individuals (95% HPDI: 32–149). The basal encounter rate (λ_0_) was 0.26 (±0.15 *SD*), and the scale parameter (σ) was 8.7 km (±2.5 km *SD*).

**Table 4:** Clouded leopard density estimates (with associated uncertainties) (per 100 km^2^) from maximum‐likelihood and Bayesian frameworks

Parameters	Maximum‐likelihood		Bayesian	
	Mean (*SE*)	95% CI	Posterior mean (*SD*)	95% CRI
σ (m)	7,594.36 (1,738.03)	4,877.16, 11,825.38	8,698.38 (2,532.86)	5,155.60, 13,976.76
*g*_0_/λ_0_	0.003 (0.001)	0.002, 0.008	0.0025 (0.0015)	0.002, 0.0055
$\hat{D}$	0.30(0.12)	0.15, 0.63	0.40 (0.10)	0.16, 0.75

CI, confidence intervals; CRI, credible intervals.

The maximum‐likelihood density estimate for clouded leopards was 0.30 (±0.12 *SE*) individuals per 100 km^2^. We ran only three models for the maximum‐likelihood analysis (Table S5). Due to computational limits and a small number of captures/recaptures (Figure S2), complex models of more than one parameter and the time‐based model were unable to run. The addition of parameters, however, did not improve the model, and we reported our results from the most parsimonious model, *g*_0_ ~ 1(Table 4). Capture probability at home range centre (*g*_0_) was estimated at 0.30 (±0.10 *SE*) per 100 km^2^, and the scale parameter σ was 7.59 km (±1.73 km *SE*). The density estimates from the two approaches were similar (Table 4). There were pockets of high‐density regions in the south (Figure S3), most of which were overlapping with high predicted site use (Figure 4).

## 4 DISCUSSION

Using bycatch from national tiger survey, we have estimated density and site‐use probability of a cryptic species, the clouded leopard, following methodologies developed in previous studies (Borah et al., 2014; Mohamad et al., 2015 for *N. nebulosa*; Sollmann et al., 2014; Haidir et al., 2013 for *N. diardi*).

The naïve site‐use probability of 13.4% was underestimated compared to the overall estimated probability of 44.8%, which was approximately three times the former. This confirmed the need to account for imperfect detection and doing so substantially improved the predictive ability of occupancy models. For a notoriously elusive species, failure to detect them when they are present at a site will induce a common source of survey bias (Linkie, Dinata, Nugroho, & Haidir, 2007). Thus, explicitly estimating detectability is important to estimate spatial distribution reliably. Probabilities of detection differed among survey areas which might be due to behavioral responses (e.g., to disturbance level in the survey areas or to the season; Tan et al., 2017). Other factors that might influence clouded leopard abundance and/or detectability include the availability of prey (Mohamad et al., 2015) and/or other large carnivores such as tigers and leopards (Tan et al., 2015). As the focus of this study is on the influence of geographical factors, the effects of interspecific interactions on the detection of the clouded leopard remain to be explored. Our study did not include covariates such as prey species and sympatric species. Obviously, an important determinant of carnivore presence is prey (Barber‐Mayer et al., 2013) and predator abundance is strongly associated with prey richness (Sandom et al., 2013). To enable sympatry within a guild, carnivores may adopt spatiotemporal segregation (Karanth et al., 2017).

**Figure 3:**
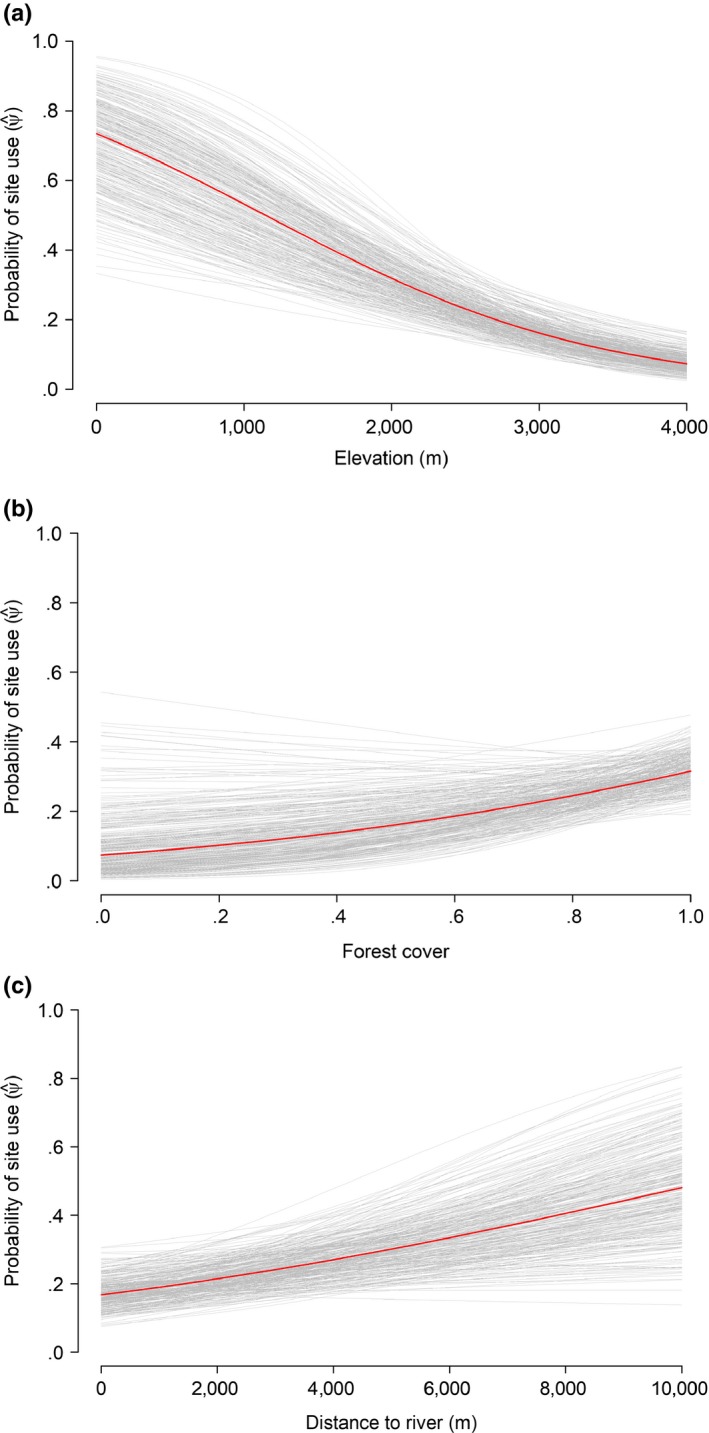
Effect of site covariates (a) elevation, (b) forest cover, and (c) distance to river on the clouded leopard site‐use probability in Bhutan ($\hat{\psi }$). Red line = posterior mean; gray lines = relationship based on a random posterior sample of 300 to visualize uncertainty (95% Bayesian credible intervals)

The best model for predicting clouded leopard site use was the Bayesian spatial model with elevation, forest cover, and distance to river covariates. In contrast to the findings of earlier studies conducted in Southeast Asia (albeit on *N. diardi* selecting higher elevation, Haidir et al., 2013; positive association of *N. nebulosa* habitat use and elevation, Ngoprasert et al., 2012; Mohamad et al., 2015; Tan et al., 2017), elevation was negatively associated with clouded leopard site use in our study (Table 3, Figure 3a). We suspect that the lowest altitude in Bhutan might be close to the highest in more tropical landscapes; hence, our finding of clouded leopards in lower elevations may not be at odds with those from earlier studies. This also suggests that clouded leopards are less likely to be found at higher elevations in Bhutan. Nonetheless, one capture was made at an altitude of 3,600 m a.s.l. (also recorded in Nepal by Can et al., *submitted*), revealing that forested habitat at high elevations may be used by clouded leopards. Elsewhere, studies have concluded that elevation was the main determinant of occupancy (Haidir et al., 2013 for *N. diardi*), while others concluded it was an indirect factor operating through an effect on prey abundance (Mohamad et al., 2015 for *N. nebulosa*). An earlier study in Bhutan suggested that elevation was not correlated with the abundance of putative prey of clouded leopards (Penjor et al., 2016), although knowledge of this felid's diet is poor (Mohamad et al., 2015).

We found that a higher percentage of forest cover in the 500 m buffer area of each camera station favored clouded leopard site use (Figure 3b). This supports our hypothesis and concurs with a previous study that demonstrated the clouded leopard preference for forest cover in Peninsular Malaysia (Tan et al., 2017). The use of forested habitat over grassland and secondary vegetation by clouded leopard has also been shown in previous studies (e.g., Austin et al., 2007). Clouded leopards are suspected to be arboreal (Rabinowitz et al., 1987) and forest‐obligate species; forest cover is thought to play a vital role in providing concealment while hunting (Nowell & Jackson, 1996). Our findings indicate that it is important to protect large tracts of forest both inside and outside protected areas. Contiguous forest cover over extensive landscapes may facilitate movement of clouded leopards, serving as corridors for dispersal. High forest cover has the potential to provide high‐value habitat that could serve as a refuge and food resources within the species’ home range (Galvez et al., 2013). High turnover of site‐use probability in corridors and areas beyond the protection offered by protected areas could reflect the importance of forest cover. Forest cover can affect the hunting success of carnivores, providing concealment (Balme, Hunter, & Slotow, 2007). Forest protection is affected by infrastructure development. Recently, Bhutan has seen the extensive construction of road network to connect all remote villages to district headquarters. In the process, the forest (largely subtropical broadleaved forest) has undergone major loss (Reddy et al., 2016). We suspect such losses are malign indicators of the decrease in important wildlife habitat.

Clouded leopards avoided habitat close to rivers (Figure 3c). Most settlements are concentrated near major rivers, probably contributing to their unattractiveness for clouded leopards (see Sunarto et al., 2012; and Tan et al., 2017). Our findings are at a grain too coarse to comment on the role of smaller rivers deeper in forests.

**Figure 4:**
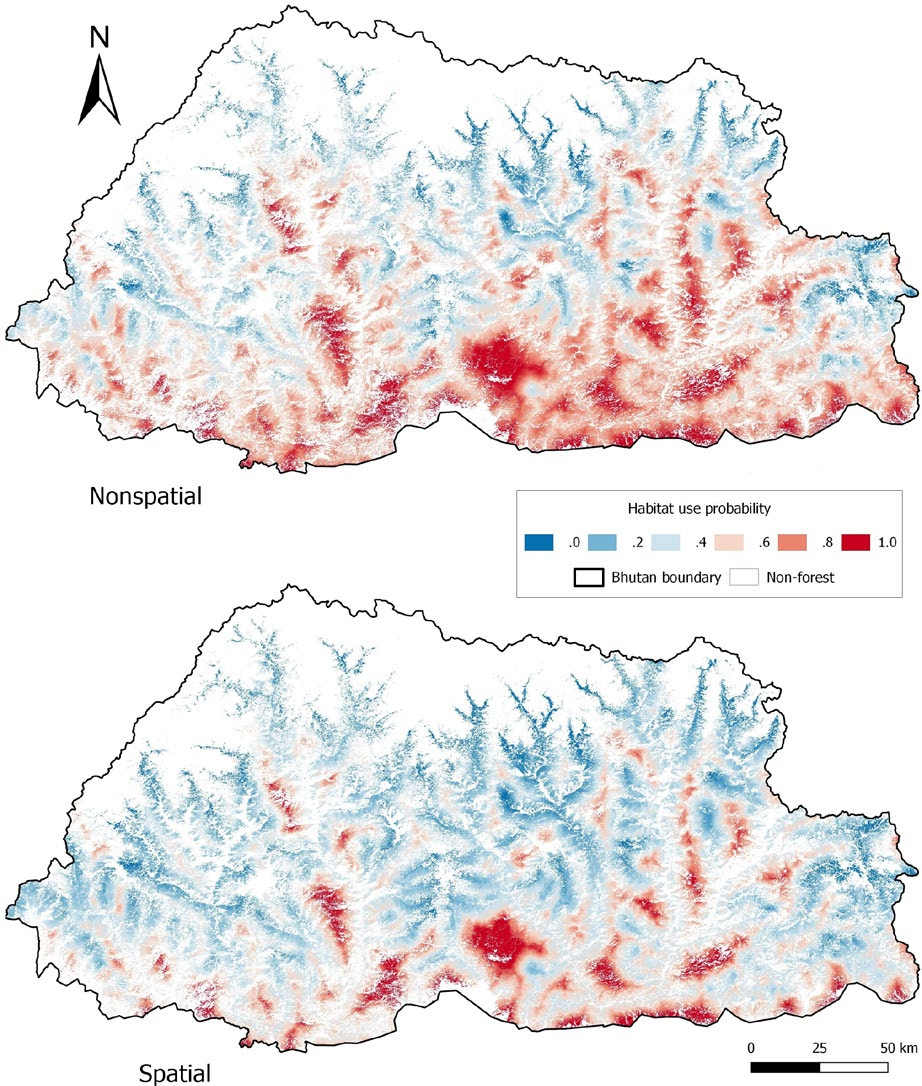
Derived clouded leopard site‐use probability in Bhutan. Top: nonspatial (maximum‐likelihood not accounting spatial autocorrelation); bottom: spatial (Bayesian accounting spatial autocorrelation; EPSG: 5266)

Failure to account for SAC resulted in overprediction, evident in the predictive map derived from the nonspatial model (Figure 4). Sites to the north and central Bhutan, with human settlement and higher elevation, were predicted to have high site use by the clouded leopard. However, based on field experiences of authors (U.P., S.W., and T.), those sites were unlikely to be used by clouded leopards. These effects showed that the lack of spatial independence in detection rates resulted in overestimated site‐use probability. The clouded leopards were moving between stations on a single occasion (as suggested by the high 8.7 km movement parameter), and hence, the detection rates of trap stations closer to one another are more similar. The spatial model, when taking into account this similarity, produced a lower site‐use probability than the nonspatial model. The large difference in occupancy estimates between spatial and nonspatial models suggests that the spatial structure is prominent; that is, we had tight concentrations of high detection rates versus low detection rates. Additionally, models accounting for SAC revealed residual spatial correlation that better captured the clouded leopard's presence. Hence, when planning space use and protection of forests, the use of the range map accounting for SAC would be more conservative and appropriate.

The density estimates from maximum‐likelihood and Bayesian approaches were similar (${\hat{D}}_{\mathrm{maximum}-\mathrm{likelihood}}=0.30/100\;{\text{km}}^{2}$ and ${\hat{D}}_{\mathrm{Bayesian}}=0.40/100\;{\text{km}}^{2}$). These estimates are comparable to, though lower than, those from produced by SCR models in previous studies (Table S5). Although little is known of clouded leopard spatial organization, female clouded leopards probably have home ranges of about 16–40 km^2^ with a core of about 5.4 km^2^ (radii of core and home range = 1.3 km and 2.99 km, respectively; Austin et al., 2007; Hearn et al.*,* 2016). The grid of 5 km × 5 km with mean spacing between stations of 2.96 km (±1.24 km *SD*) can certainly miss such cores, leaving “holes” in the grid (Noss et al., 2012). The camera traps would be placed further apart for a survey grid designed for tigers than for clouded leopards. This spacing would help in minimizing the violation of the assumption of occupancy analysis that the “detection of the species at a site is independent of detecting the species at any other sites.” However, for density estimation, this spacing would introduce “holes” and likely yield conservative estimates. Our analyses also do not account for biases such as a possible difference in detecting males and females on and off trails (Balme, Hunter, & Slotow, 2009; Wegge, Pokheral, & Jnawali, 2004). Such methodological biases are widely recognized and deserve further research (Foster & Harmsen, 2012; see also Sollmann et al., 2011; Broekhuis & Gopalaswamy, 2016). Our study provides the first density estimates of the clouded leopard in Bhutan. This information sets an important benchmark for clouded leopard monitoring programs and underscores the rarity of this species to prompt conservation planning.

### 4.1 Methodological considerations

Although we successfully used the same dataset to estimate both density and occupancy (site use), there are certain assumptions underlying SCR and occupancy modeling that must be adhered to strictly in order to reduce bias in estimates. SCR requires adequate captures and recaptures at multiple sites, while occupancy, on the other hand, requires spatial independence between sites. Notwithstanding the risk of violating the underlying assumptions, we reconciled these requirements by explicitly modeling occupancy (site use) using the restricted spatial regression (RSR) method incorporating both detection probability and correcting for spatial autocorrelation (see Johnson et al., 2013 for details). The RSR model had two main effects: a decrease in the average probability of site use and narrowing of the width of confidence intervals of the covariates (Table 3). The posterior predictive loss criterion (PPLC) favored the spatial model over the nonspatial model, although the difference was small. The performance of PPLC for hierarchical models is still unknown and therefore should be interpreted with care. It is analogous to Akaike information criterion (AIC) for model selection but does not show whether the model fits the data well (Broms et al., 2014).

### 4.2 Management implications

A suite of species–habitat relationships for clouded leopard was identified, some of which accords well with the results of previous studies, whereas others are new. For example, we found a negative relationship between site use and elevation, the opposite of previous studies (Tan et al., 2017 for *N. nebulosa*; Haidir et al., 2013 for *N. diardi*), probably due to differences in altitude ranges between studies. We recommend, therefore, that future studies on clouded leopards in Bhutan place camera traps at lower altitudes. Our use of bycatch information derived from the large‐scale tiger survey illustrates the great potential for additional returns on investment in camera‐trap studies (Mohamad et al., 2015; Sollmann et al., 2014). The predictive mapping indicated that forests inside and outside protected areas were equally suitable for the clouded leopard (Figure S1). Habitat suitability was high in the low‐altitude areas of the southern region (Table 5). Clouded leopards have considerable potential as ambassador species, fostering the protection of sympatric carnivores (Asiatic golden cat *Catopuma temminckii*, marbled cat *Pardofelis marmorata,* and leopard cat *Prionailurus bengalensis*; Singh & Macdonald, 2017). Although growing human footprint is a cause for concern, we did not find evidence that it exerts pressure on the current distribution of clouded leopard. Nevertheless, our finding of an effect of distance to river may indicate an indirect effect of disturbance on habitat selection. Management should seek to minimize loss of forest cover and identify, at a finer scale, the limiting factors influencing site use in particular areas. The predictive spatial occupancy map, together with the density map, also revealed many isolated but seemingly optimal sites for the clouded leopard conservation beyond protected areas (Figures S1 and S3). We hope these maps will prove useful for future land‐use and conservation planning.

**Table 5:** Predicted mean site‐use probabilities of the clouded leopard in Bhutan

Site	Mean	*SD*
Wangchuck Centennial National Park (WCNP)	**0.362**	0.056
Sakteng Wildlife Sanctuary (SWS)	**0.370**	0.055
Royal Manas National Park (RMNP)	0.513	0.062
Phipsoo Wildlife Sanctuary (PWS)	0.485	0.021
Phrumsengla National Park (PNP)	**0.420**	0.053
Jomotshangkha Wildlife Sanctuary (JWS)	0.501	0.064
Jigme Singye Wangchuck National Park (JSWNP)	0.454	0.095
Jigme Khesar Strict Nature Reserve (JKSNR)	**0.423**	0.055
Jigme Dorji National Park (JDNP)	**0.378**	0.064
Bumdelling Wildlife Sanctuary (BWS)	**0.401**	0.063
Bumthang Division	**0.400**	0.053
Gedu Division	0.461	0.066
Mongar Division	0.472	0.056
Paro Division	**0.402**	0.046
Samtse Division	**0.445**	0.058
Sarpang Division	0.524	0.076
Samdrupjongkhar Division	0.481	0.066
Trashigang Division	**0.445**	0.056
Thimphu Division	**0.390**	0.040
Tsirang Division	0.482	0.072
Wangdue Division	0.452	0.076
Zhemgang Division	0.458	0.059

Bold values indicate that the 95% CI of the mean predicted site use is below and not overlapping the overall average of 0.448. Divisions are nonprotected areas. Refer to Supporting information Figure S1 for site location.

## CONFLICT OF INTEREST

None declared.

## AUTHORS’ CONTRIBUTION

U.P., D.W.M., and C.K.W.T. conceived the ideas and designed analyses; S.W. designed and coordinated the field survey; T., with support from field staff, collected data; U.P. analyzed the data; U.P. led the writing of the manuscript. D.W.M. and C.K.W.T. provided input on the manuscript. All authors contributed critically to the drafts and gave final approval for publication.

## DATA ACCESSIBILITY

All the data used in the analyses will be made publicly available with the supporting information. Few information (such as coordinates) due to sensitive nature (data from national tiger survey) will not be available on public domain.
