# Supplementary material for: Identifying important conservation areas for the clouded leopard Neofelis nebulosa in a mountainous landscape: Inference from spatial modeling techniques
Source: Ecol Evol. 2018 Apr 2;8(8):4278–91. doi: 10.1002/ece3.3970 (PMC5916301; doi:10.1002/ece3.3970)
Supplement: Supplementary file 1 [file ECE3-8-4278-s001.docx]

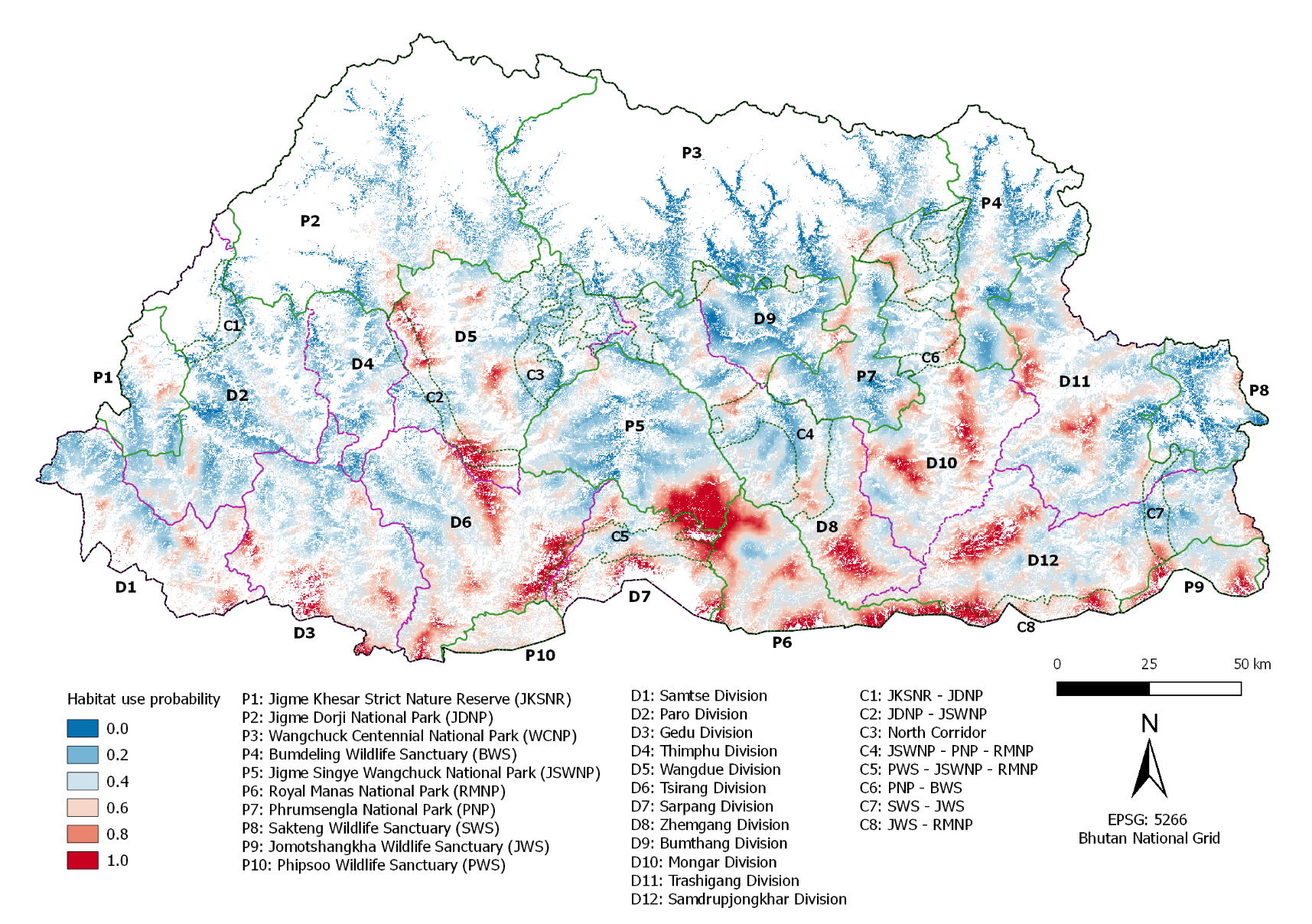


**Figure S1.** Site use probability of clouded leopard across different management regimes (P – protected area, D – outside protected area and C – corridors; see legend for details; also refer to Table 5 for details).
