# Supplementary material for: Identifying important conservation areas for the clouded leopard Neofelis nebulosa in a mountainous landscape: Inference from spatial modeling techniques
Source: Ecol Evol. 2018 Apr 2;8(8):4278–91. doi: 10.1002/ece3.3970 (PMC5916301; doi:10.1002/ece3.3970)
Supplement: Supplementary file 2 [file ECE3-8-4278-s002.docx]

Recapture

Individual ID

**Figure S2.** Recaptures of 19 (identified) individual clouded leopards used in SCR analysis.
