# Supplementary figures and images for: Identifying important conservation areas for the clouded leopard Neofelis nebulosa in a mountainous landscape: Inference from spatial modeling techniques
Source: Ecol Evol. 2018 Apr 2;8(8):4278–91. doi: 10.1002/ece3.3970 (PMC5916301; doi:10.1002/ece3.3970)

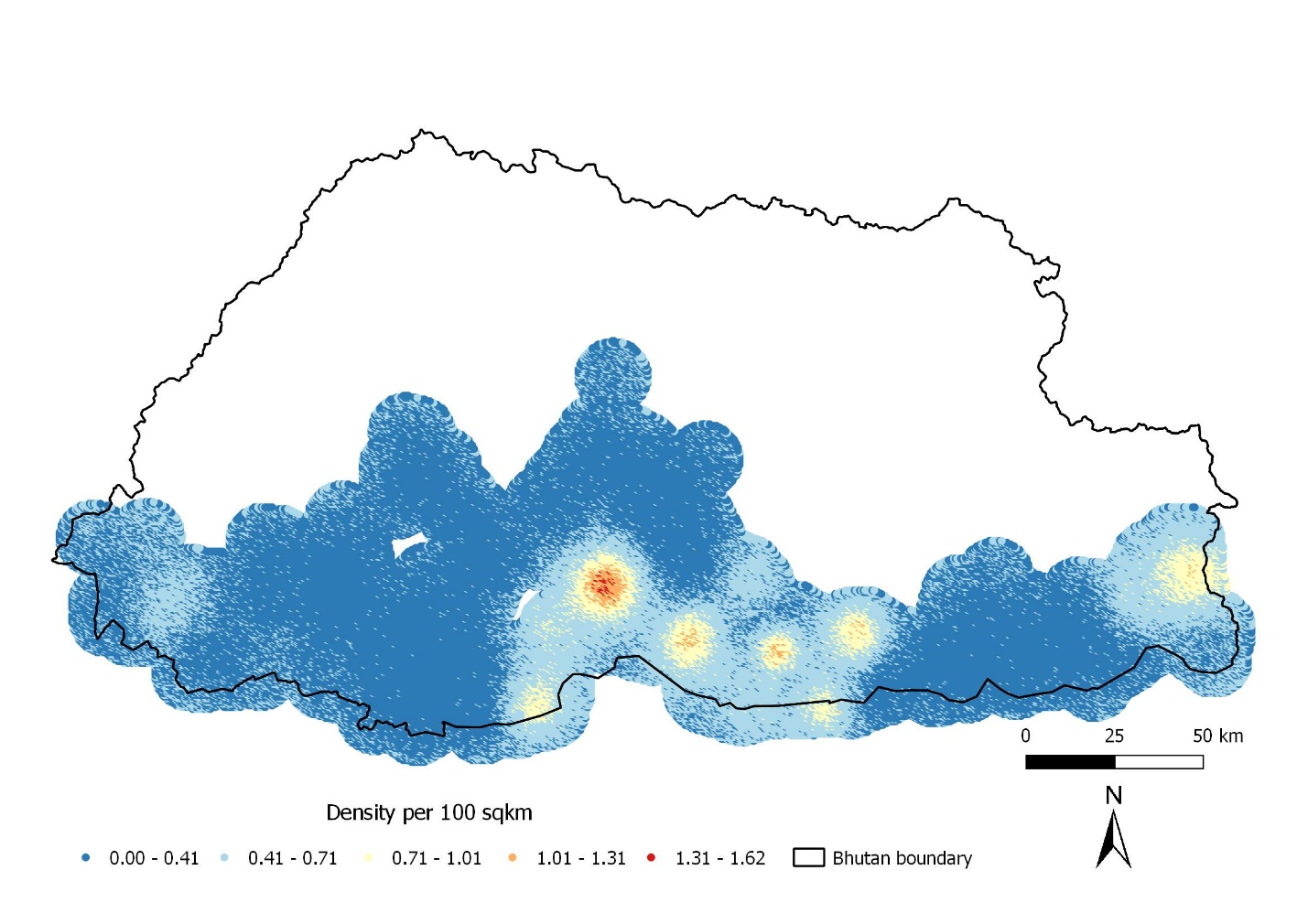


**Figure S3.** Density of clouded leopards across southern Bhutan. (EPSG: 5266)

Supplement: Supplementary file 3 [file ECE3-8-4278-s003.docx]
