# Supplementary material for: Identifying important conservation areas for the clouded leopard Neofelis nebulosa in a mountainous landscape: Inference from spatial modeling techniques
Source: Ecol Evol. 2018 Apr 2;8(8):4278–91. doi: 10.1002/ece3.3970 (PMC5916301; doi:10.1002/ece3.3970)
Supplement: Supplementary file 4 [file ECE3-8-4278-s004.docx]

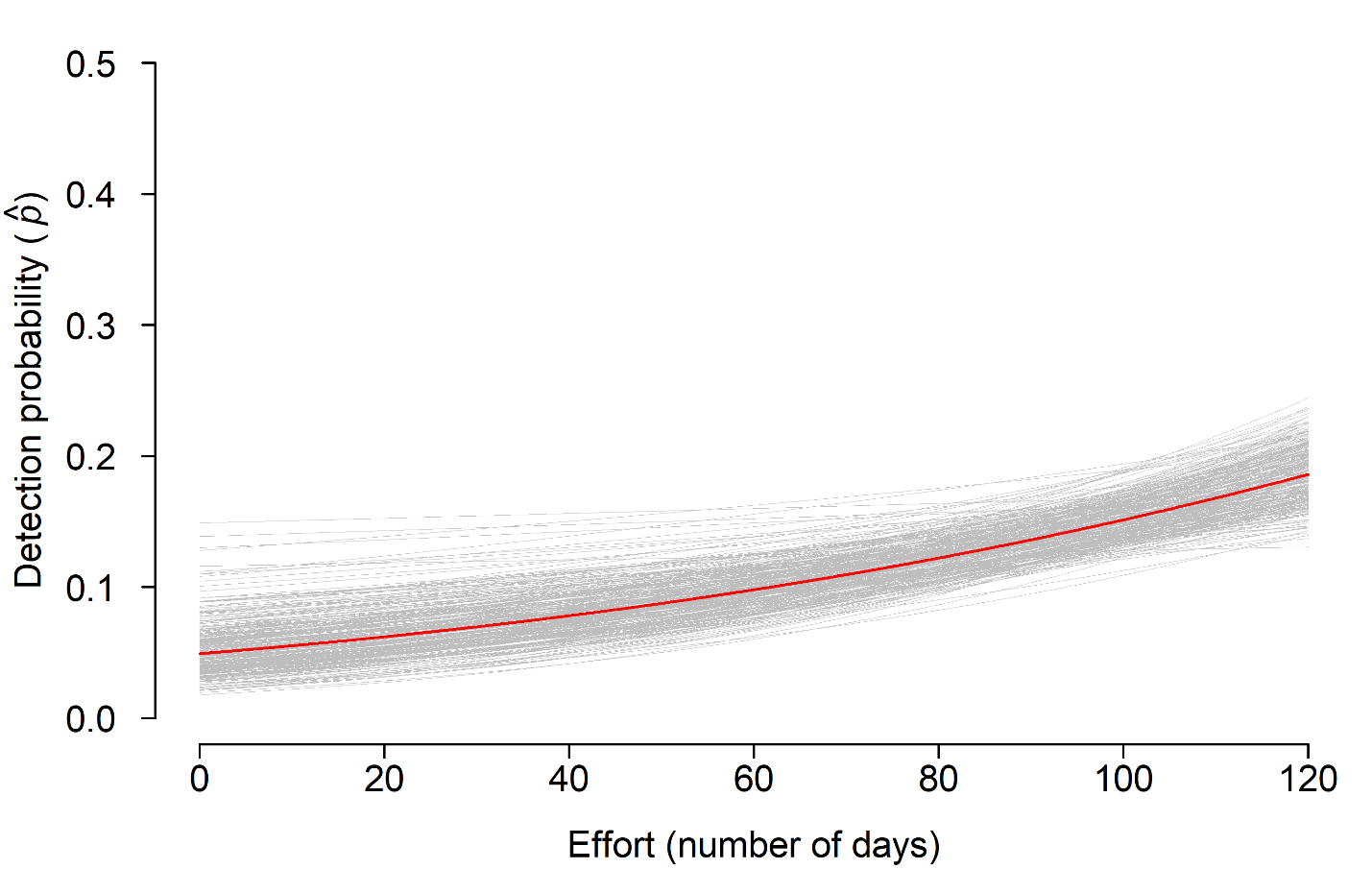


**Figure S4.** Detection probability ($\hat{p}$) as a function of number of active camera trap days (effort) for clouded leopard site use probability in Bhutan (red line = posterior mean, grey line = 95% credible intervals from 300 samples).
