# Supplementary material for: Identifying important conservation areas for the clouded leopard Neofelis nebulosa in a mountainous landscape: Inference from spatial modeling techniques
Source: Ecol Evol. 2018 Apr 2;8(8):4278–91. doi: 10.1002/ece3.3970 (PMC5916301; doi:10.1002/ece3.3970)
Supplement: Supplementary file 5 [file ECE3-8-4278-s005.docx]

**Table S1.** Pearson’s correlation matrix of the continuous site covariates. Pairs of covariates were considered highly correlated when r>|0.6| (bold). Site covariates tested were: elevation (ELEVATION), distance to logged forest (LOG), Global Forest Change with four different threshold values (GFC30, GFC50, GFC75, GFC90), distance to river (RIV), distance to roads (ROA), distance to settlement (SET), slope (SLO), and Vegetation Continuous Field (VCF), aspect (ASP) and distance to protected area (PA).

|  | ASP | ELE | G30 | G50 | G75 | G90 | LOG | PA | RIV | ROA | SET | SLO |  |
| --- | --- | --- | --- | --- | --- | --- | --- | --- | --- | --- | --- | --- | --- |
| ELE | 0.005 |  |  |  |  |  |  |  |  |  |  |  |  |
| G30 | 0.014 | -0.398 |  |  |  |  |  |  |  |  |  |  |  |
| G50 | 0.001 | -0.464 | **0.964** |  |  |  |  |  |  |  |  |  |  |
| G75 | 0.049 | -0.394 | **0.768** | **0.85** |  |  |  |  |  |  |  |  |  |
| G90 | -0.027 | -0.353 | 0.358 | 0.426 | **0.612** |  |  |  |  |  |  |  |  |
| LOG | -0.015 | -0.501 | 0.012 | 0.04 | 0.007 | -0.009 |  |  |  |  |  |  |  |
| PA | -0.019 | 0.119 | -0.036 | -0.024 | -0.047 | -0.076 | -0.132 |  |  |  |  |  |  |
| RIV | 0.049 | 0.103 | 0.015 | 0.012 | 0.021 | 0.045 | -0.193 | 0.059 |  |  |  |  |  |
| ROA | 0.015 | 0.071 | -0.108 | -0.094 | -0.039 | -0.017 | 0.359 | -0.172 | -0.167 |  |  |  |  |
| SET | -0.004 | 0.162 | -0.175 | -0.184 | -0.186 | -0.172 | 0.125 | -0.168 | -0.04 | 0.47 |  |  |  |
| SLO | 0.024 | 0.159 | 0.026 | -0.012 | 0.058 | 0.196 | -0.094 | 0.049 | -0.15 | 0.113 | -0.154 |  |  |
| VCF | -0.019 | -0.2 | **0.74** | **0.717** | **0.661** | 0.394 | -0.133 | 0.042 | 0.043 | -0.02 | -0.215 | 0.285 |  |
|  |  |  |  |  |  |  |  |  |  |  |  |  |  |
