# Supplementary material for: Identifying important conservation areas for the clouded leopard Neofelis nebulosa in a mountainous landscape: Inference from spatial modeling techniques
Source: Ecol Evol. 2018 Apr 2;8(8):4278–91. doi: 10.1002/ece3.3970 (PMC5916301; doi:10.1002/ece3.3970)
Supplement: Supplementary file 6 [file ECE3-8-4278-s006.docx]

**Table S2.** Clouded leopard univariate occupancy (ψ) models. AIC_c_ akaike’s information criterion corrected for small sample size. ΔAIC_c_ relative difference in AIC_c_ values compared with the top ranked model, AIC_c_wt AIC_c_ weight, K number of parameters. Site covariates tested were: elevation (ELEVATION), distance to logged forest (LOG), Global Forest Change with four different threshold values (GFC30, GFC50, GFC75, GFC90), distance to river (RIV), distance to roads (ROA), distance to settlement (SET), slope (SLO), and Vegetation Continuous Field (VCF), aspect (ASP) and distance to protected area (PA).

| **Model** | **K** | **AIC_c_** | **Δ AIC_c_** | **AIC_c_Wt** | **Cum. Wt** | **-2logLik** |
| --- | --- | --- | --- | --- | --- | --- |
| *p*(.)*ψ*(ELE) | 3 | 1340.42 | 0.00 | 0.62 | 0.62 | -667.20 |
| *p*(.)*ψ*(GFC90) | 3 | 1343.47 | 3.05 | 0.14 | 0.76 | -668.72 |
| *p*(.)*ψ*(GFC50) | 3 | 1343.50 | 3.08 | 0.13 | 0.89 | -668.74 |
| *p*(.)*ψ*(GFC75) | 3 | 1343.99 | 3.57 | 0.00 | 1.00 | -668.98 |
| *p*(.)*ψ*(GFC30) | 3 | 1350.39 | 9.97 | 0.00 | 1.00 | -672.18 |
| *p*(.)*ψ*(VCF) | 3 | 1359.97 | 19.55 | 0.00 | 1.00 | -676.97 |
| *p*(.)*ψ*(RIV) | 3 | 1361.88 | 21.46 | 0.00 | 1.00 | -677.93 |
| *p*(.)*ψ*(PA) | 3 | 1367.49 | 27.07 | 0.00 | 1.00 | -680.73 |
| *p*(.)*ψ*(.) | 2 | 1372.91 | 32.49 | 0.00 | 1.00 | -684.45 |
| *p*(.)*ψ*(ROA) | 3 | 1374.45 | 34.03 | 0.00 | 1.00 | -684.21 |
| *p*(.)*ψ*(LOG) | 3 | 1374.74 | 34.32 | 0.00 | 1.00 | -684.36 |
| *p*(.)*ψ*(ASP) | 3 | 1374.90 | 34.48 | 0.00 | 1.00 | -684.43 |
| *p*(.)*ψ*(SLO) | 3 | 1374.91 | 34.49 | 0.00 | 1.00 | -684.44 |
| *p*(.)*ψ*(SET) | 3 | 1374.92 | 34.50 | 0.00 | 1.00 | -684.44 |
