# Supplementary material for: Identifying important conservation areas for the clouded leopard Neofelis nebulosa in a mountainous landscape: Inference from spatial modeling techniques
Source: Ecol Evol. 2018 Apr 2;8(8):4278–91. doi: 10.1002/ece3.3970 (PMC5916301; doi:10.1002/ece3.3970)
Supplement: Supplementary file 7 [file ECE3-8-4278-s007.docx]

**Table S3.** Chi-square probability (χ^2^*p*) and over dispersion statistic (*ĉ*) results of the MacKenzie and Bailey (2004) goodness of fit test for clouded leopard occupancy models with different collapsing day- periods. Detection covariate was number of days a camera trap station was active for during each sampling occasion (EFFORT). Occupancy covariates tested were: elevation (ELEVATION), distance to logged forest (LOG), Global Forest Change with four different threshold values (GFC30, GFC50, GFC75, GFC90), distance to river (RIV), distance to roads (ROA), distance to settlement (SET), slope (SLO), and Vegetation Continuous Field (VCF), aspect (ASP) and distance to protected area (PA). Subset of 120 sampling days of the dataset and total of 849 camera-trap stations were used for analysis.

| **Collapsing period** | **χ^2^*p*** | ***ĉ*** |
| --- | --- | --- |
| 10-days sampling occasions | 0.278 | 0.61 |
| 12-days sampling occasions | 0.133 | 1.22 |
| 15-days sampling occasions | 0.206 | 0.74 |
