# Supplementary material for: Identifying important conservation areas for the clouded leopard Neofelis nebulosa in a mountainous landscape: Inference from spatial modeling techniques
Source: Ecol Evol. 2018 Apr 2;8(8):4278–91. doi: 10.1002/ece3.3970 (PMC5916301; doi:10.1002/ece3.3970)
Supplement: Supplementary file 8 [file ECE3-8-4278-s008.docx]

**Table S4.** Beta coefficients with standard errors and 95% confidence intervals (CI) of the explanatory variables included in the likelihood-based occupancy models of clouded leopard site use probability in Bhutan. Covariates with weak effect and 95% CI including zero.

| **Covariates** | $\hat{\boldsymbol{\beta}}$ **coefficients** | | **95% CI** |
| --- | --- | --- | --- |
|  | **Mean** | **SE** |  |
| Logged forest | -0.232 | 0.193 | -0.610, 0.146 |
| Distance to road | 0.215 | 0.170 | -0.118, 0.548 |
| Slope | 0.160 | 0.152 | -0.138, 0.459 |
| Distance to Protected Area | -0.147 | 0.163 | -0.468, 0.173 |
| Aspect | 0.043 | 0.153 | -0.258, 0.343 |
