# Supplementary material for: Identifying important conservation areas for the clouded leopard Neofelis nebulosa in a mountainous landscape: Inference from spatial modeling techniques
Source: Ecol Evol. 2018 Apr 2;8(8):4278–91. doi: 10.1002/ece3.3970 (PMC5916301; doi:10.1002/ece3.3970)
Supplement: Supplementary file 9 [file ECE3-8-4278-s009.docx]

**Table S5**. Model comparison in secr package. *secr0*: base model with constant detection probability, *secrb*: behavioral response present and *secrh*: individual heterogeneity in detection. AICc Akaike’s information criterion corrected for small sample sizes, ∆AICc relative difference between AICc of subsequent models compared to top ranked model, AICcWt AICc weight and npar is the number of parameters.

| **Model** | | **Detection function** | **npar** | **logLik** | **AIC_c_** | **ΔAIC_c_** | **AIC_c_Wt** |
| --- | --- | --- | --- | --- | --- | --- | --- |
| *secr0* | g0~1 | Half-normal | 2 | -189.545 | 383.840 | 0.000 | 0.689 |
| *secrb* | g0~b | Half-normal | 3 | -188.693 | 384.986 | 1.819 | 0.278 |
| *secrh* | g0~h2 | Half-normal | 4 | -189.209 | 389.274 | 6.107 | 0.033 |
