# Supplementary material for: Identifying important conservation areas for the clouded leopard Neofelis nebulosa in a mountainous landscape: Inference from spatial modeling techniques
Source: Ecol Evol. 2018 Apr 2;8(8):4278–91. doi: 10.1002/ece3.3970 (PMC5916301; doi:10.1002/ece3.3970)
Supplement: Supplementary file 10 [file ECE3-8-4278-s010.docx]

**Table S6.** Comparison of density estimates using various methods in south-east Asia

| **Species** | **Country** | **Source** | **Year** | $\hat{\boldsymbol{D}}$**(SE) 100km^-2^** | **method** |
| --- | --- | --- | --- | --- | --- |
| mainland | Malaysia | Davies & Payne | 1982 | 1/4 km^2^ or 25/100km^2^ | Pugmark |
| Sunda | Malaysia | Wilting et al., | 2006 | 9 (95% CI 8-17) | Pugmark |
| Sunda | Borneo (Malaysia) | Wilting et al., | 2012 | 1 (0.84±0.42-1.04±0.58) | SECR (B) |
| Sunda | Borneo (Malaysia) | Brodie & Giordano | 2012 | 0.8 (95% CI 0.2-2.6) | SECR(MLE) |
| Sunda | Indonesia | Cheyne et al., | 2013 | 0.72-4.41 | CAPTURE |
| mainland | India | Borah et al., | 2013 | 4.73±1.43 | SECR (MLE) |
| Sunda | Sumatra | Sollmann et al., | 2014 | 0.39-1.28 | SECR (B) |
| mainland | Malaysia | Mohamad et al., | 2015 | 1.83±0.61 (Royal Belum)  3.46±1.00 (Temengor) | SECR (MLE) |
| mainland | Malaysia | Vaca et al., | 2015 (unp.) | 2.28±1.11 (B)  2.9±1.8 (MLE) | SECR (B & MLE) |
| mainland | Bhutan | This study | 2017 | 0.30±0.12 (MLE)  0.40±0.10 (B) | SECR (B & MLE) |
