# Supplementary material for: Identifying important conservation areas for the clouded leopard Neofelis nebulosa in a mountainous landscape: Inference from spatial modeling techniques
Source: Ecol Evol. 2018 Apr 2;8(8):4278–91. doi: 10.1002/ece3.3970 (PMC5916301; doi:10.1002/ece3.3970)
Supplement: Supplementary file 11 [file ECE3-8-4278-s011.docx]

**Table S7.** Details for camera trap survey. Effort is the total number of days where camera traps were active.

| Survey area | Number of stations | Mean number of days deployed(SD) | Clouded leopard captures | Effort |
| --- | --- | --- | --- | --- |
| Royal Manas National Park | 69 | 101.4(48.5) | 43 | 6593 |
| Jigme Singye Wangchuck National Park | 62 | 78.2(32) | 42 | 4849 |
| Phipsoo Wildlife Sanctuary | 21 | 131.9(55.9) | 19 | 2550 |
| Jigme Dorji National Park | 38 | 38.5(30.6) | 1 | 1463 |
| Phrumsengla National Park | 26 | 66.8(41.8) | 6 | 1738 |
| Bumdeling Wildlife Sanctuary | 29 | 103.1(47.1) | 10 | 2989 |
| Sakteng Wildlife Sanctuary | 22 | 75.7(62.2) | 0 | 1665 |
| Jigme Khesar Strict Nature Reserve | 15 | 66.4(33.1) | 0 | 996 |
| Wangchuck Centenniel National Park | 31 | 25.3(14.3) | 0 | 783 |
| Gedu Division | 35 | 74.4(51.9) | 2 | 2603 |
| Sarpang Division | 39 | 85.4(28.6) | 18 | 3331 |
| Samtse Division | 8 | 95.2(25.3) | 1 | 762 |
| Samdrupjongkhar Division | 78 | 74.5(40.1) | 12 | 5809 |
| Wangdue Division | 81 | 58.3(39.5) | 4 | 4724 |
| Tsirang Division | 38 | 108.4(49.5) | 8 | 4120 |
| Zhemgang Division | 102 | 71.1(35.9) | 16 | 7248 |
| Paro Division | 40 | 64.9(33.7) | 0 | 2597 |
| Thimphu Division | 21 | 58.2(60.4) | 0 | 1223 |
| Mongar Division | 32 | 57.729.2 () | 4 | 1789 |
| Trashigang Division | 25 | 88.6(32.8) | 6 | 2215 |
| Bumthang Division | 37 | 72.8(39.7) | 0 | 2692 |
| Total | 849 |  |  | 62739 |
